# Synthesis and crystal structures of *N*,2,4,6-tetra­methyl­anilinium tri­fluoro­methane­sulfonate and *N*-iso­propyl­idene-*N*,2,4,6-tetra­methyl­anilinium tri­fluoro­methane­sulfonate

**DOI:** 10.1107/S2056989024003438

**Published:** 2024-04-26

**Authors:** John W. Stewart, Elena M. Irons, Giovanna Osorio Abanto, Matthias Zeller, Curtis M. Zaleski, Daniel P. Predecki

**Affiliations:** a Department of Chemistry and Biochemistry, Shippensburg University, Shippensburg, PA 17257, USA; b Department of Chemistry, Purdue University, West Lafayette, IN 47907, USA; Illinois State University, USA

**Keywords:** crystal structure, iminium ion, ammonium ion, tri­fluoro­methane­sulfonate, tri­fluoro­methane­sulfonate, 2–4,6-tri­methyl­aniline, methyl­ation, *N*,2,4,6-tetra­methyl­anilinium, *N*-iso­propyl­idene-*N*,2,4,6-tetra­methyl­anilinium

## Abstract

Two 2,4,6-tri­methyl­aniline-based trifuloro­methane­sulfonate (tri­fluoro­methane­sulfonate) salts were synthesized and characterized by single-crystal X-ray diffraction. *N*,2,4,6-Tetra­methyl­anilinium tri­fluoro­methane­sulfonate, [C_10_H_14_NH_2_^+^][CF_3_O_3_S^−^] (**1**), was synthesized *via* methyl­ation of 2,4,6-tri­methyl­aniline. *N*-Iso­propyl­idene-*N*,2,4,6-tetra­methyl­anilinium tri­fluoro­meth­ane­sulfonate, [C_13_H_20_N^+^][CF_3_O_3_S^−^] (**2**), was synthesized in a two-step reaction where the imine, *N*-iso­propyl­idene-2,4,6-tri­methyl­aniline, was first prepared *via* a dehydration reaction to form the Schiff base, followed by methyl­ation using methyl tri­fluoro­methane­sulfonate to form the iminium ion. In compound **1**, both hydrogen bonding and π–π inter­actions form the main inter­molecular inter­actions. The primary inter­action is a strong N—H⋯O hydrogen bond with the oxygen atoms of the tri­fluoro­methane­sulfonate anions bonded to the hydrogen atoms of the ammonium nitro­gen atom to generate a one-dimensional chain. The [C_10_H_14_NH_2_^+^] cations form dimers where the benzene rings form a π–π inter­action with a parallel-displaced geometry. The separation distance between the calculated centroids of the benzene rings is 3.9129 (8) Å, and the inter­planar spacing and ring slippage between the dimers are 3.5156 (5) and 1.718 Å, respectively. For **2**, the [C_13_H_20_N^+^] cations also form dimers as in **1**, but with the benzene rings highly slipped. The distance between the calculated centroids of the benzene rings is 4.8937 (8) Å, and inter­planar spacing and ring slippage are 3.3646 (5) and 3.553 Å, respectively. The major inter­molecular inter­actions in **2** are instead a series of weaker C—H⋯O hydrogen bonds [C⋯O distances of 3.1723 (17), 3.3789 (18), and 3.3789 (18) Å], an inter­action virtually absent in the structure of **1**. Fluorine atoms are not involved in strong directional inter­actions in either structure.

## Abstract 2

Two aniline-based tri­fluoro­methyl­sulfonate (tri­fluoro­methane­sulfonate) salts, *N*,2,4,6-tetra­methyl­anilinium tri­fluoro­methane­sulfonate and *N*-iso­propyl­idene-*N*,2,4,6-tetra­methyl­anilinium tri­fluoro­methane­sulfonate, were synthesized and characterized by single-crystal X-ray diffraction.

## 1. Chemical context

Aniline, the simplest aromatic amine, was first isolated by Otto Unverdorben in 1826 by the destruction of indigo dye. Since its discovery, aniline-based compounds have been extensively utilized as precursors to dyestuffs, pharmaceuticals, polymers, explosives, and industrial feedstocks (Travis, 2007 ▸). Of relevance to this work, *N*-methyl­aniline has been used to synthesize a variety of poly-*N*-methyl­aniline materials that function as electrodes, batteries, and nanocomposite sorbents to remove metal ions from solution (Lü *et al.*, 2014 ▸). In addition, N-substituted anilines, including *N*-methyl-2,4,6-tri­methyl­aniline, have been used in the preparation of α-amino diazo­ketones, which have been used as precursors in the synthesis of HIV inhibitors (Castoldi *et al.*, 2018 ▸).

Condensation of aniline with aldehydes and ketones leads to the formation of Schiff bases otherwise known as imines, of which the primary functional group is a carbon–nitro­gen double bond (Tsuchimoto *et al.*, 1973 ▸; Layer, 1963 ▸). Addition of an extra atom or group to the imine nitro­gen leads to the formation of iminium ions. Iminium ions have been identified as versatile inter­mediates in traditional organic chemistry, such as in the Knoevenagel and Mannich reactions and have also been utilized in the synthesis of natural products and pharmaceuticals (Erkkilä *et al.*, 2007 ▸; Böhme *et al.*, 1976 ▸). Herein, we report synthesis and characterization of two anilinium-based triflate salts, *N*,2,4,6-tetra­methyl­anilinium tri­fluoro­methane­sulfonate, [C_10_H_14_NH_2_^+^][CF_3_O_3_S^−^] (**1**), and *N*-iso­propyl­idene-*N*,2,4,6-tetra­methyl­anilinium tri­fluoro­methane­sulfonate, [C_13_H_20_N^+^][CF_3_O_3_S^−^] (**2**). 
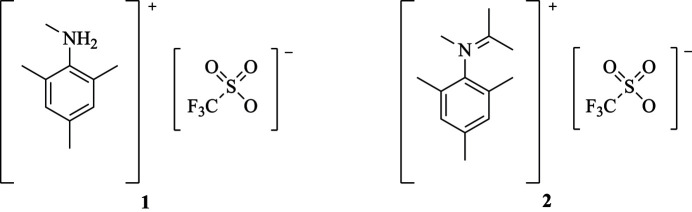


## 2. Structural commentary

**Figure 1:**
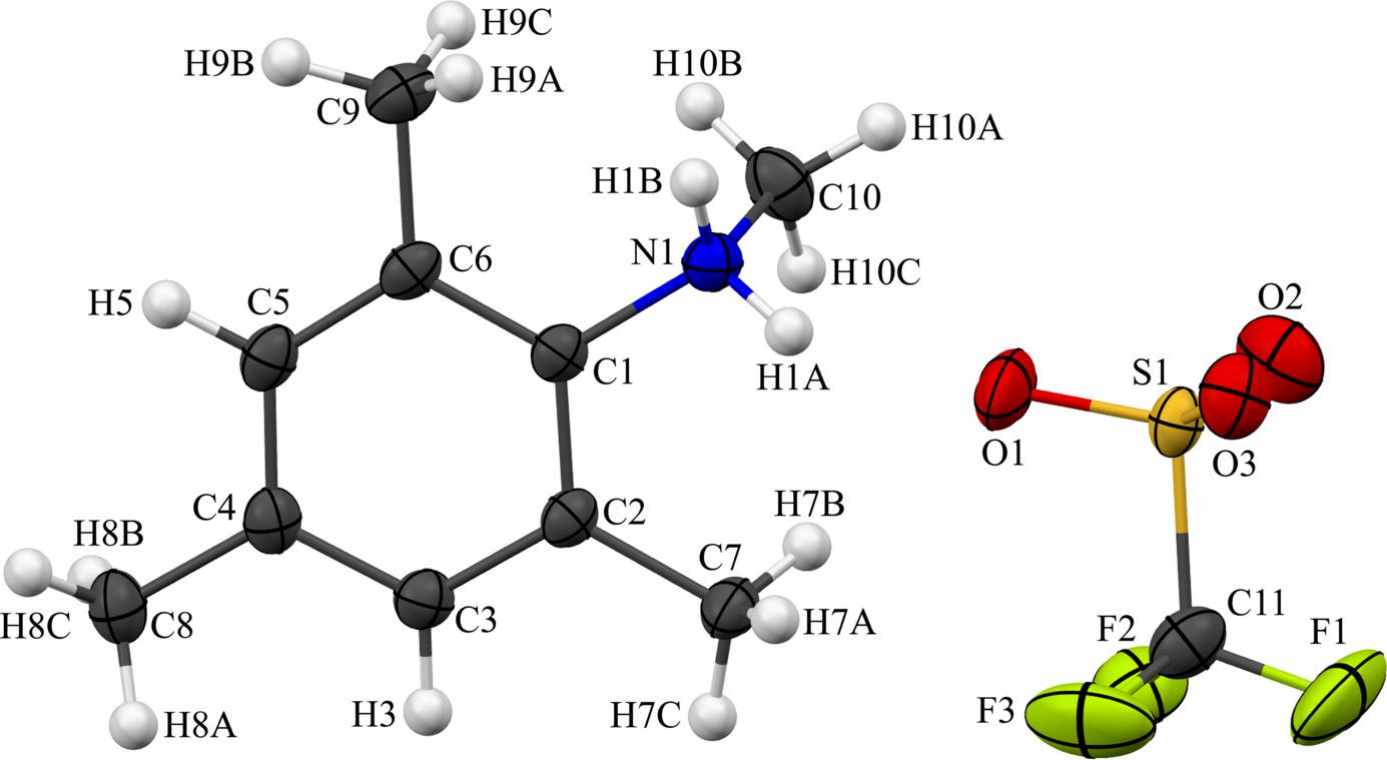
The single-crystal X-ray structure of *N*,2,4,6-tetra­methyl­anilinium tri­fluoro­methane­sulfonate, [C_10_H_14_NH_2_^+^] [CF_3_O_3_S^−^] (**1**). Displacement ellipsoids are at the 50% probability level. Color scheme: gray – carbon, blue – nitro­gen, red – oxygen, yellow – fluorine, orange – sulfur, and white – hydrogen. All figures were generated with the program *Mercury* (Macrae *et al.*, 2020 ▸).

**Figure 2:**
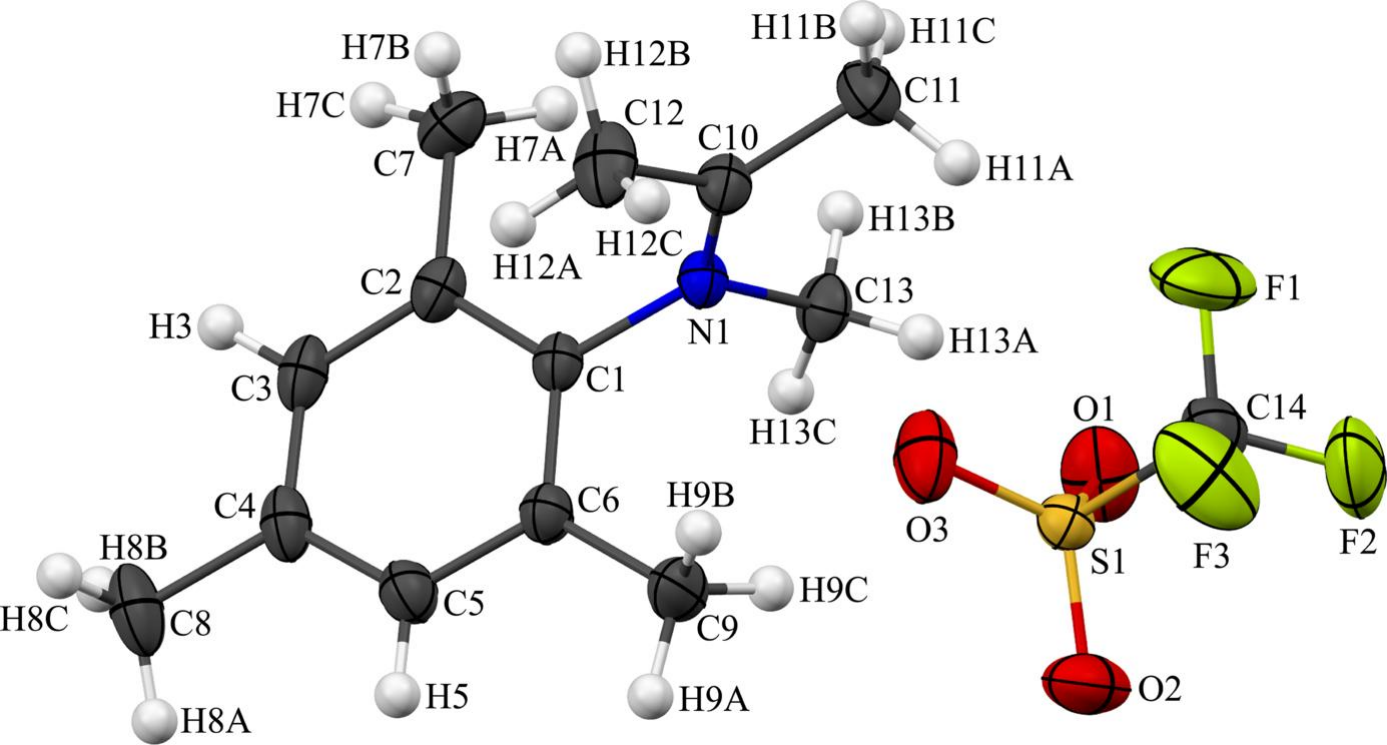
The single-crystal X-ray structure of *N*-methyl­iso­propyl­idene-*N*,2,4,6-tetra­methyl­anilinium tri­fluoro­methane­sulfonate, [C_13_H_20_N^+^] [CF_3_O_3_S^−^] (**2**). See Fig. 1 ▸ for additional display details.

**Figure 3:**
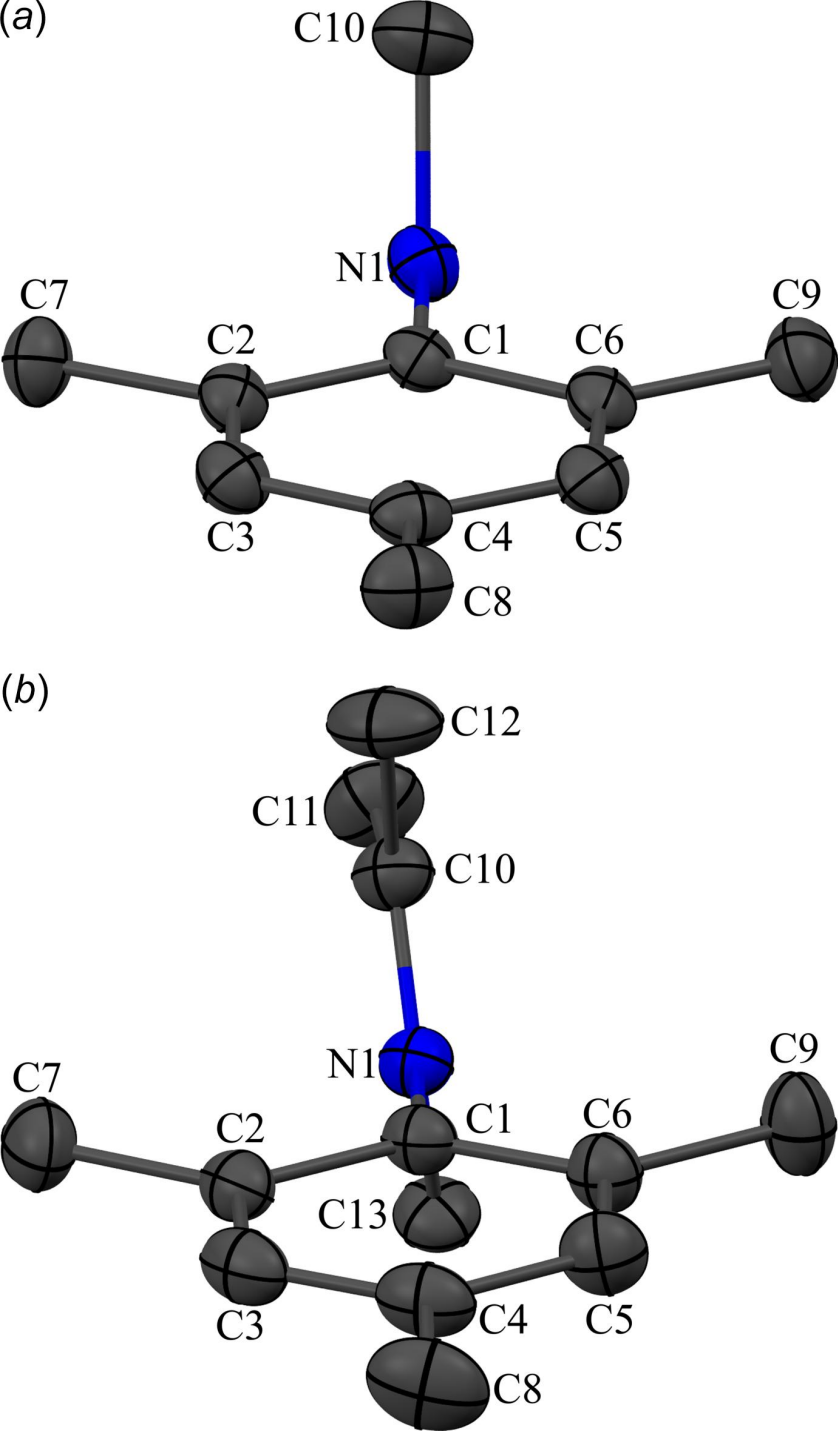
The organic functional groups bound to nitro­gen atom are approximately orthogonal to the ring of the 2,4,6-tri­methyl­phenyl group for both (*a*) **1** and (*b*) **2**. For clarity, the hydrogen atoms have been omitted. See Fig. 1 ▸ for additional display details.

Both compounds **1** and **2** are ionic compounds based on cations of a 2,4,6-tri­methyl­anilinium unit with functionalization of the amine group and the anion tri­fluoro­methane­sulfonate (*i.e.* triflate). For **1**, a secondary ammonium ion results from bonds to a 2,4,6-tri­methyl­phenyl ring, a methyl group, and two hydrogen atoms (Fig. 1 ▸). The hydrogen atoms of the ammonium nitro­gen atom form hydrogen bonds with the oxygen atoms of neighboring triflate anions. For **2**, the iminium ion consists of an iso­propyl­idene group (nitro­gen atom double bonded to a carbon atom attached to two methyl groups) with a 2,4,6-tri­methyl­phenyl ring and a methyl group also attached to the nitro­gen (Fig. 2 ▸). As there are no hydrogen atoms on the iminium nitro­gen atom, the organic cation of **2** does not form any classical hydrogen bonds. The 2,4,6-tri­methyl­phenyl groups in both **1** and **2**, and the iso­propyl­idene group in **2** are, as expected, nearly planar with r.m.s. deviations from planarity (including the nitro­gen atom in the defined planes) of only 0.0263, 0.0111 and 0.0200 Å, respectively. In both compounds, the carbon functional groups of the nitro­gen atom lie approximately perpendicular to the tri­methyl­phenyl ring (Fig. 3 ▸). For **1**, the angle between the calculated mean plane of the methyl group (defined as C1, N1 and C10) and the mean plane of the aniline ring (N1 and C1–C6) is 89.71 (9)°, while for **2**, the angle between the mean plane of the iso­propyl­idene and methyl groups (C1, N1, and C10–C13) and the mean plane of the aniline ring (N1, C1–C6) is 85.15 (4)° (Table 1 ▸).

## 3. Supra­molecular features

The dominant inter­molecular forces in **1** consist of strong N—H⋯O hydrogen bonding (Table 2 ▸) and π–π stacking inter­actions (Table 3 ▸), while in **2** no classical hydrogen bonds are present and π–π inter­actions are highly slipped. Instead, inter­actions in **2** are governed by a series of weak C—H⋯O/F inter­actions (Table 4 ▸; listed H⋯O/F distances are up to 2.70 Å). Similar C—H⋯O inter­actions are also present in **1** but they are much less pronounced; the C—H⋯O distances and angles indicate that they are more likely dispersion (*i.e.* van der Waals) inter­actions rather than weak directional hydrogen bonds. For both compounds, C—H⋯F and C—H⋯π inter­actions are very weak and not well defined (Tables 2 ▸–4 ▸ ▸).

**Figure 4:**
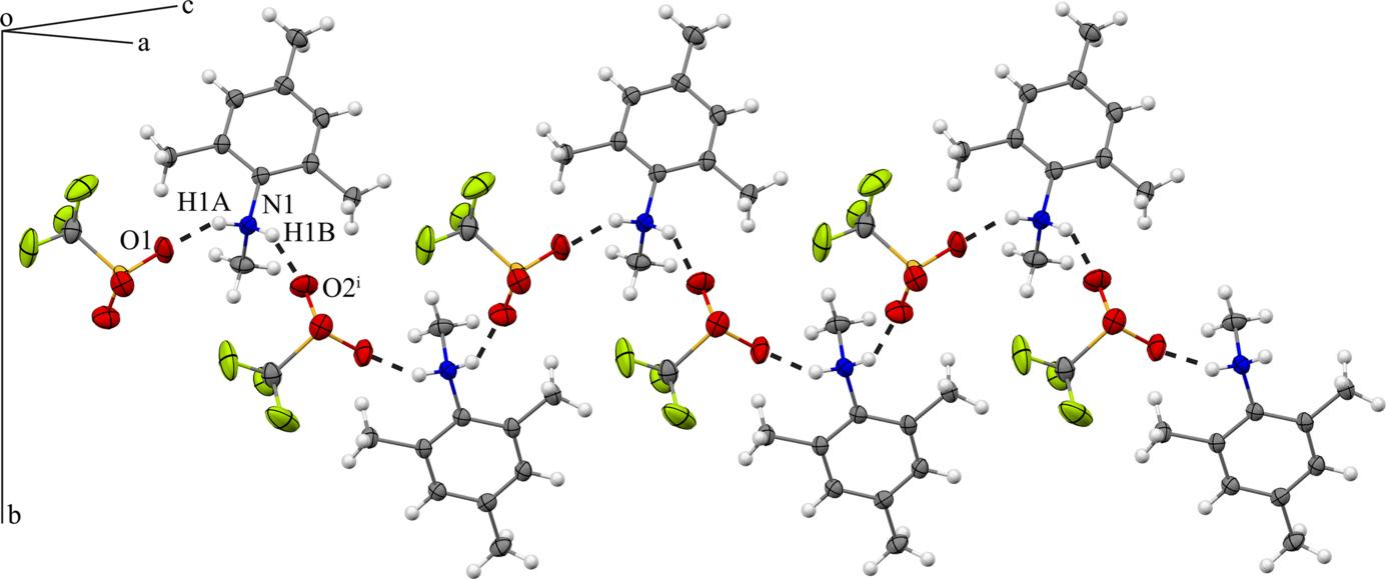
Inter­molecular hydrogen bonding in **1** between the ammonium hydrogen atoms and the tri­fluoro­methane­sulfonate oxygen atoms. The hydrogen bonding results in a one-dimensional chain that extends in the *ac* plane. For clarity, only the atoms involved in the hydrogen bonding are labeled. See Fig. 1 ▸ for additional display details. [Symmetry code: (i) *x* + 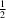 , −*y* + 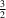 , *z* + 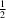 .]

**Figure 5:**
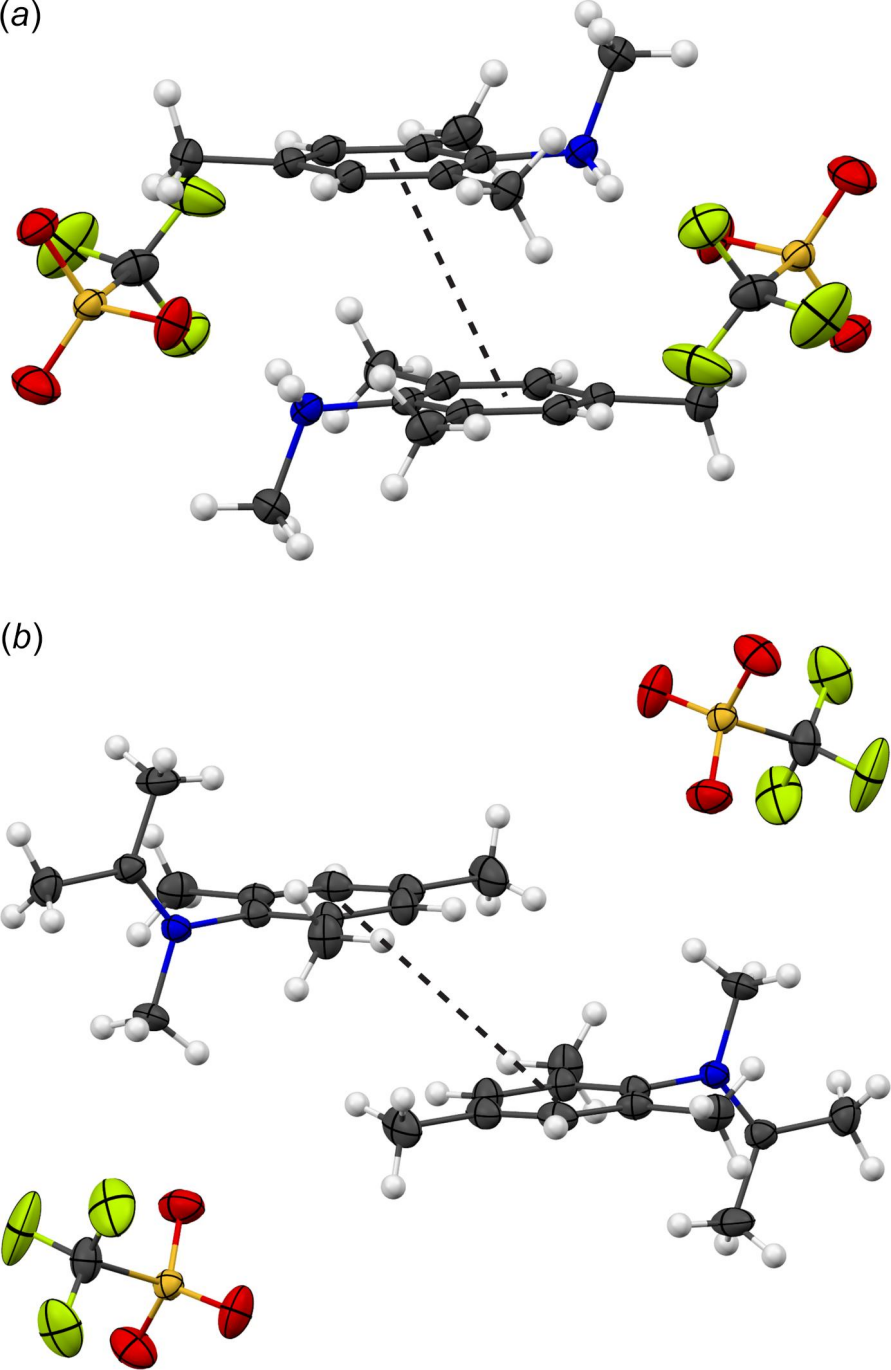
The organic cations of (*a*) **1** and (*b*) **2** form dimers that are related by a crystallographic inversion center and have inter­molecular π–π inter­actions in a parallel-displaced geometry (black dotted lines). See Fig. 1 ▸ for additional display details.

For **1**, the principal directional inter­actions are the N—H⋯O hydrogen bonds. Both ammonium hydrogen atoms are hydrogen bonded to an oxygen atom of neighboring triflate anions (Fig. 4 ▸; Table 2 ▸). One triflate anion is located on either side of the ammonium nitro­gen atom. The hydrogen-bonding arrangement leads to a one-dimensional chain that extends in the *ac* plane and is propagated by the *n*-glide plane at (*x*, 0.75, *z*). In addition, the organic cations form dimers with the 2,4,6-tri­methyl­phenyl rings arranged in a parallel-displaced geometry where the 2,4,6-tri­methyl­phenyl rings are offset relative to each other. The cations that make up the dimers are symmetry-related by inversion so that the ammonium groups are opposite of each other, likely to avoid Coulombic repulsions (Fig. 5 ▸). The distance between the calculated centroids of the benzene rings in each dimer is 3.9129 (8) Å, and the inter­planar spacing and ring slippage are 3.5156 (5) and 1.718 Å, respectively [determined with *PLATON* (Spek, 2020 ▸); Table 3 ▸].

**Figure 6:**
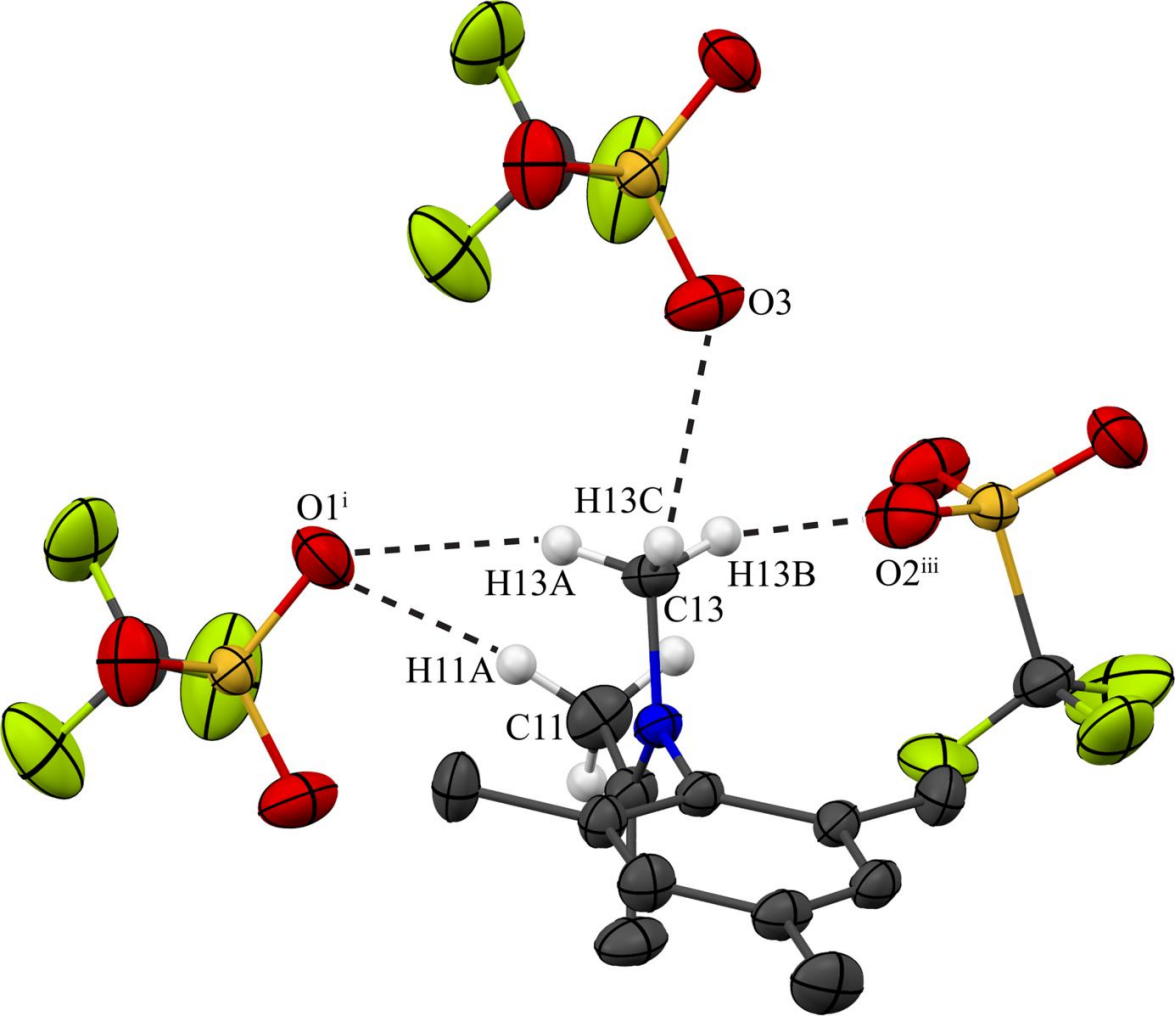
Inter­molecular hydrogen bonding in **2** between the methyl hydrogen atoms of the [C_13_H_20_N^+^] cation and the oxygen atoms of three different tri­fluoro­methane­sulfonate anions. For clarity, only the hydrogen atoms on the methyl groups involved in the hydrogen bonding are shown and only the atoms involved in the hydrogen bonding are labeled. See Fig. 1 ▸ for additional display details. [Symmetry codes: (i) *x* + 1, *y*, *z*; (iii) *x* + 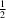 , −*y* + 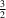 , *z* + 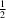 .]

In **2**, the organic cations also form inversion-related dimers, but the rings are highly slipped (Fig. 5 ▸) with respect to each other and π–π inter­action, if present at all, is limited to just the outermost atoms C4 and C5. The distance between the calculated centroids of the benzene rings in each dimer is 4.8937 (8) Å, and inter­planar spacing and ring slippage are 3.3646 (5) and 3.553 Å, respectively (Table 3 ▸). In the absence of classical hydrogen bonding as well as significant π–π inter­actions, other weak inter­molecular forces become dominant in the structure of **2**. Most obvious are a series of weaker C—H⋯O inter­actions (Table 4 ▸). Most important are the hydrogen-bond-like inter­actions that involve the iminium methyl group (C13) being hydrogen bonded to oxygen atoms of three different triflate anions (Fig. 6 ▸). This methyl group is directly bonded to the nitro­gen atom and carries the largest partial positive charge, inducing formation of charge-assisted bonds that are unusually short for C—H⋯O inter­actions with C⋯O distances of 3.1723 (17), 3.3789 (18), and 3.3789 (18) Å (Desiraju & Steiner, 2001 ▸). The iso­propyl­idene methyl group (C11) also does exhibit another unusually short C—H⋯O bond [C⋯O distance of 3.477 (2) Å] (Fig. 6 ▸). Each [C_13_H_20_N^+^] cation is hydrogen bonded to three triflate anions, and each triflate anion is hydrogen bonded to three organic cations, thereby generating a two-dimensional network of C—H⋯O inter­actions with layers extending perpendicular to the *b*-axis direction. The resulting layers inter­act with each other solely *via* dispersion inter­actions.

Both the centroid distance between the benzene rings and the ring slippage distance are longer for **2** than **1**. However, the values for **2** are more aligned with the distances for 1,3,5-tri­methyl­benzene, *i.e*. mesitylene. In the crystal structure of deuterated-1,3,5-tri­methyl­benzene (SOPLAL01; Ibberson *et al.*, 2007 ▸) the mol­ecules also form long π–π inter­actions with a parallel-displaced geometry, and the distance between the calculated centroids of neighboring benzene rings is 4.634 Å with a ring slippage of 2.850 Å (Table 3 ▸). Moreover, the longer distances of **2** are comparable to the centroid and ring slippage distances for a series of 2,4,6-tri­methyl­anilinium cations with various counter-anions (Table 3 ▸).

## 4. Database survey

A survey of the Cambridge Structural Database (CSD version 5.45, update November 2023; Groom *et al.*, 2016 ▸) for secondary ammonium cations with a 2,4,6-tri­methyl­phenyl group, as in **1**, yielded five entries (EDUWAD, HIBFOO, HIBFUU, HIBGAB and QARJUQ). Two of the cations have two 2,4,6-tri­methyl­phenyl groups bound to the ammonium nitro­gen atom but with different counter-anions, penta­fluoro­benzene­sulfonate (HIBFOO; Sakakura *et al.*, 2007 ▸) or 4-methyl­benzene­sulfonate (HIBGAB; Sakakura *et al.*, 2007 ▸). A related secondary ammonium cation binds to one 2,4,6-tri­methyl­phenyl group and one 2,6-di­phenyl­phenyl group and the counter-anion is a penta­fluoro­benzene­sulfonate (HIBFUU; Sakakura *et al.*, 2007 ▸). The last two entries also only contain one 2,4,6-tri­methyl­phenyl group on the ammonium nitro­gen atom. In one structure (EDUWAD; Ikhile & Bala, 2012 ▸), an ethyl-2-formamido-2,4,6-tri­methyl­benzene group is bound to the ammonium nitro­gen atom and chloride serves as the counter-anion. The other structure (QARJUQ; Latham *et al.*, 2012 ▸) is a zwitterion with a 3-methyl­butan-2-yl-carbamoyl­benzene­sulfonate acting as the second group bound to the ammonium nitro­gen atom. Lastly, a biphenyl system with two secondary ammonium nitro­gen atoms (CATZEG; Li *et al.*, 2022 ▸) is similar to **1**. As in **1**, the 1,1′ biphenyl system has an ammonium nitro­gen attached to the carbon atom in the 4 and 4′ positions of the benzene rings and on each benzene ring two methyl groups are located on carbon atoms adjacent (3,3′ and 5,5′ positions, respectively) to the carbon atom with the ammonium nitro­gen atom. In addition, the ammonium nitro­gen atoms bind to a methyl group as in **1**. A comparison of the angle between the mean planes of the functional groups and of the aniline ring reveal that the angles generally do not approach 90° as in **1** (Table 1 ▸; the angles were measured between mean planes defined in a similar manner as for **1** in the *Structural commentary* section). The angles range from *ca* 50 to 77° for the five structures with a 2,4,6-tri­methyl­phenyl group attached to the ammonium nitro­gen atom. For these structures, the bulkiness of the groups opposite the 2,4,6-tri­methyl­phenyl groups may prevent the angle being close to 90°. In **1**, the group opposite to the 2,4,6-tri­methyl­phenyl group is a smaller methyl group. For the 1,1′-biphenyl system (CATZEG) the angle (*ca* 85°) is closer to 90° likely due to the two phenyl rings nearly lying in the same plane and the presence of a smaller methyl group.

A survey for compounds containing an iminium nitro­gen atom with a 2,4,6-tri­methyl­phenyl ring and with a double-bonded carbon atom bound to two additional carbon groups yielded only one entry (JIFFAI; Kremláček *et al.*, 2018 ▸). Like **2**, the iminium nitro­gen atom is bound to a methyl group and a 2,4,6-tri­methyl­phenyl group and the counter-anion is triflate. Unlike **2**, substitution on the carbon atom of the iminium double bond consists of a methyl group and a bulky 3-methyl-2-(2,4,6-tri­methyl­phen­yl)-2*H*-indazol-7-yl group. Comparison of the equivalent angle between the mean planes of the functional groups and the aniline ring to that of **2** reveals that the angle (*ca* 83°) deviates more from 90° than that of **2** (Table 1 ▸). In a related structure (RAVBIC; Chen *et al.*, 2017 ▸), substitution on the carbon atom of the iminium double bond consists of a hydrogen atom and a {2-[(hydrox­yl)(meth­oxy)methyl­idene]-4-meth­oxy-4-oxo}{[tris­(penta­fluoro­phen­yl)]boron} group. In addition, the iminium nitro­gen is bound to a methyl group. Also, this compound is a zwitterion instead of a triflate salt with the borate providing the negative charge. In regard to the angle between the mean planes of the functional groups and the aniline ring, there is an even larger deviation (*ca* 80°), likely due to the bulky {2-[(hydrox­yl)(meth­oxy)methyl­idene]-4-meth­oxy-4-oxo}{[tris­(penta­fluoro­phen­yl)]boron} group (Table 1 ▸).

## 5. Synthesis and crystallization

**Synthetic Materials**

Methyl tri­fluoro­methane­sulfonate (98%) and 4 Å mol­ecular sieves (8–12 mesh) were purchased from Sigma-Aldrich. Anhydrous diethyl ether (99%, ACS Grade) and 2,4,6-tri­methyl­aniline (97%) were purchased from Thermo Scientific. Acetonitile-*d*_3_ (99 atom %D) was purchased from Acros Organics. Chloro­form (99.5%) was purchased from Fisher Scientific. Acetone (99.5%) was purchased from VWR Chemicals. Chloro­form was dried over 4 Å mol­ecular sieves (8–12 mesh) prior to use. All other chemicals were used as received and without further purification.

***N*** **,2,4,6-tetra­methyl­anilinium tri­fluoro­methane­sulfonate (1)** (29.2%). Dried chloro­form (2.0 mL), methyl tri­fluoro­methane­sulfonate (0.290 mL, 2.4 mmol, 1.1 eq) and a stir bar were added to a dry 10 mL round-bottom flask flushed with nitro­gen gas. 2,4,5-tri­methyl­aniline (0.3200 g, 2.4 mmol, 1 eq) was dissolved in 1.0 mL of dried chloro­form and added dropwise to the flask with stirring over ice with the resulting solution appearing clear and colorless. The flask was allowed to stir for 15 min over ice and an additional 30 min at room temperature. House vacuum was then used to remove the solvent, leaving behind an off-white powder. The powder was redissolved in 1–2 mL of dried chloro­form with 10 drops of anhydrous diethyl ether. Clear, colorless crystals were grown in 1–2 days by slow evaporation of the solvent at room temperature. The clear crystals (0.2090 g, 29.2%) were vacuum filtered and washed with 5.0 mL of anhydrous diethyl ether. A portion of the crystals were separated for X-ray diffraction analysis with the remaining sample being analyzed as follows: m.p. 429.2–430.5 K; IR (ATR) ν_max_ 3082 cm^−1^ (N^+^—H stretch), 1610 cm^−1^ (N^+^—H bend); ^1^H-NMR (CD_3_CN, 80 MHz): δ 2.29 (*s*, 3 H), 2.40 (*s*, 6 H), 3.02 (*t*, *J* = 2.83 Hz, 3 H), 7.04 (*s*, 1 H); ^13^C{1H}-NMR (CD_3_CN, 80 MHz): δ 17.24, 20.68, 37.43, 131.50, 131.76, 132.18, 140.84.

***N*** **-iso­propyl­idene-*N*,2,4,6-tetra­methyl­anilinium tri­fluoro­methane­sulfonate (2)** (20.7% over two steps). Synthesis was carried out using a two-step process. In step one, *N*-iso­propyl­idene-2,4,6-tri­methyl­aniline was synthesized utilizing the procedure published by Tsuchimoto *et al.* (1973 ▸). Anhydrous diethyl ether (35.1 mL) was added to a 60 mL amber glass bottle followed by 2,4,6-tri­methyl­aniline (1.5241 g, 11.3 mmol, 1 eq), acetone (1.00 mL, 13.6 mmol, 1.2 eq), and 4 Å mol­ecular sieves (15.6 g) resulting in a clear, slightly brown solution. The bottle was moved to the fridge and reaction progress was monitored by observing the disappearance of the 2,4,6-tri­methyl­aniline peaks by ^1^H-NMR. After about 4 days, the sieves were removed by gravity filtration and washed with three 10 mL portions of anhydrous diethyl ether. The resulting solution was rotary evaporated yielding a clear, colorless oil (1.4867 g, 75.3%). The oil was purified by fractional short path vacuum distillation (338.8–340.3 K at 1 mm Hg) to yield three clear liquid fractions, with the second fraction (0.4686 g, 23.7% post-distillation) being used in the next step. IR (ATR) v­_max_ 1670 cm^−1^ (C=N stretch); ^1^H-NMR (CD_3_CN, 80MHz): δ 1.58 (*s*, 3 H), 1.90 (*s*, 6 H), 2.15 (*s*, 3 H), 2.21 (*s*, 3 H), 6.82 (*s*, 2 H); ^13^C{1H}-NMR (CD_3_CN, 80 MHz): δ 17.98, 20.86, 27.73, 126.61, 129.33, 132.33, 147.69, 169.49.

In step two, dried chloro­form (2.0 mL), methyl tri­fluoro­methane­sulfonate (0.324 mL, 2.9 mmol, 1.1 eq) and a stir bar were added to a dry 10 mL round-bottom flask flushed with nitro­gen. *N*-iso­propyl­idene-2,4,6-tri­methyl­aniline (0.4686 g, 2.7 mmol, 1 eq) was dissolved in dried chloro­form (1 mL) and added dropwise to the flask with stirring over ice resulting a clear and colorless solution. The flask was allowed to stir for 15 min over ice and an additional 30 min at room temperature. Upon completion, house vacuum was used to remove the solvent, leaving behind an off-white powder. The powder was then redissolved in 1–2 mL of dried chloro­form with 10 drops of anhydrous diethyl ether. White crystals were grown in 1–2 days by slow evaporation of the solvent at room temperature. The white crystals (0.7931 g, 87.4%) were vacuum filtered and washed with 5.0 mL of anhydrous diethyl ether. A portion of the crystals were separated for X-ray diffraction analysis with the remaining sample being analyzed as follows: m.p. 359.0–360.4 K; IR (ATR) ν_max_ 1648 cm^−1^ (C=N stretch); ^1^H-NMR (CD_3_CN, 80 MHz): δ 2.16 (*s*, 6 H), 2.25 (*s*, 3 H), 2.33 (*s*, 3 H), 2.74 (*s*, 3 H), 3.77 (*s*, 3 H), 7.12 (*s*, 2 H); ^13^C{1H}-NMR (CD_3_CN, 80 MHz): δ 16.98, 20.81, 24.93, 26.09, 45.42, 131.01, 131.90, 141.50, 196.51.

**Physical Methods**

The ^1^H and ^13^C chemical shifts were reported in ppm (δ) and referenced to CD_3_CN. All NMR spectra were recorded at 299.7 K on a Magritek Spinsolve 80 (Malvern, PA USA). Proton and carbon spectra were operated at 80.98 MHz and 20.36 MHz, respectively, with a field strength of 1.88 Tesla. Spectra were processed using MNova software Ver. 14.3.3 (Mestrelab Research, Escondido, CA USA). Infrared spectroscopy was performed using a Nicolet iS5 FTIR spectrometer (Thermo Electron North America LLC) outfitted with a diamond crystal ATR accessory and Omnic software Omnic version 9.2.98.

## 6. Refinement

Crystal data, data collection and structure refinement details are summarized in Table 5 ▸. Hydrogen atoms were placed in calculated positions and refined as riding on their carrier atoms with C—H distances of 0.95 Å for *sp* ^2^ carbon atoms, 0.98 Å for methyl carbon atoms, and 0.91 Å for ammonium nitro­gen atoms. Methyl hydrogen atoms were allowed to rotate but not to tip to best fit the experimental electron density. The *U*_iso_ values for hydrogen atoms were set to a multiple of the value of the carrying carbon atom or nitro­gen atom (1.2 times for *sp* ^2^-hybridized carbon atoms and the nitro­gen atom or 1.5 times for methyl carbon atoms).

## Supplementary Material

Crystal structure: contains datablock(s) 1, 2. DOI: 10.1107/S2056989024003438/ej2004sup1.cif

Structure factors: contains datablock(s) 1. DOI: 10.1107/S2056989024003438/ej20041sup2.hkl

Structure factors: contains datablock(s) 2. DOI: 10.1107/S2056989024003438/ej20042sup3.hkl

Supporting information file. DOI: 10.1107/S2056989024003438/ej20041sup4.cml

Supporting information file. DOI: 10.1107/S2056989024003438/ej20042sup5.cml

CCDC references: 2349130, 2349129

Additional supporting information:  crystallographic information; 3D view; checkCIF report

## Figures and Tables

**Table 1:** Angle Between the Mean Plane of the Organic Functional Groups and the Mean Plane of the Aniline Ring Angles were determined with *SHELXL* (for **1** and **2**; Sheldrick, 2015*b* ▸) or *Mercury* (for comparison compounds; Macrae *et al.*, 2020 ▸).

Compound	Angle (°)	CSD Reference Code	CCDC Deposition Number
*N*,2,4,6-Tetra­methyl­anilinium tri­fluoro­methane­sulfonate (**1**)	89.71 (9)	This Work	
*N*-Iso­propyl­idene-*N*,2,4,6-tetra­methyl­anilinium tri­fluoro­methane­sulfonate (**2**)	85.15 (4)	This Work	
Dimesityl­ammonium penta­fluoro­benzene­sulfonate	49.87 and 55.67	HIBFOO	297281
Dimesityl­ammonium tosyl­ate	49.49 and 52.91	HIBGAB	604748
Oxonium *N*-(2,6-di­phenyl­phen­yl)mesityl­ammonium bis­(penta­fluoro­benzene­sulfonate)	55.19	HIBFUU	297282
(2,4,6-Tri­methyl­phen­yl){2-[*N*-(2,4,6-tri­methyl­phen­yl)formamido]­eth­yl}ammonium chloride	75.48	EDUWAD	878245
(*S*)-2-{[1-(Mesityl­ammonio)-3-methyl­butan-2-yl]carbamo­yl}benzene­sulfonate	76.75	QARJUQ	843836
*catena*-[*N* ^4^,*N* ^4^′,3,3′,5,5′-hexa­meth­yl[1,1′-biphen­yl]-4,4′-bis­(aminium) hexa­kis­(μ-bromo)­dilead(II)]	85.42	CATZEG	2145329
*N*-Methyl-1-[3-methyl-2-(2,4,6-tri­methyl­phen­yl)-2*H*-indazol-7-yl]-*N*-(2,4,6-tri­methyl­phen­yl)ethan-1-iminium tri­fluoro­methane­sulfonate	82.92	JIFFAI	1842546
{2-[(Hy­droxy)(meth­oxy)methyl­idene]-4-meth­oxy-*N*-methyl-4-oxo-*N*-(2,4,6-tri­methyl­phen­yl)butan-1-iminiumato}[tris­(penta­fluoro­phen­yl)]boron	80.47	RAVBIC	1504471

**Table 2:** Hydrogen-bond geometry (Å, °) for **1** [Chem scheme1]

*D*—H⋯*A*	*D*—H	H⋯*A*	*D*⋯*A*	*D*—H⋯*A*
N1—H1*A*⋯O1	0.91	1.92	2.7687 (16)	154
N1—H1*B*⋯O2^i^	0.91	1.94	2.7669 (16)	150
C8—H8*C*⋯O1^ii^	0.98	2.63	3.453 (2)	142
C8—H8*C*⋯O3^ii^	0.98	2.69	3.6438 (19)	164
C8—H8*A*⋯O3^iii^	0.98	2.55	3.504 (2)	165
C9—H9*A*⋯O2^i^	0.98	2.63	3.333 (2)	129

Symmetry codes: (i) 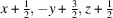 ; (ii) 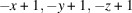 ; (iii) 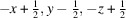 .

**Table 3:** π–π Inter­actions with parallel-displaced geometry (Å) Distances determined with *PLATON* (Spek, 2020 ▸).

Compound/cation	Anion	Benzene ring centroid–centroid distance	Inter­planar spacing	Slippage	CSD Refcode	CCDC Deposition Number
*N*,2,4,6-Tetra­methyl­anilinium	CF_3_SO_3_^−^	3.9129 (8)	3.5156 (5)	1.718	This Work (**1**)	
*N*-Iso­propyl­idene-*N*,2,4,6-tetra­methyl­anilinium	CF_3_SO_3_^−^	4.8937 (8)	3.3646 (5)	3.553	This Work (**2**)	
1,3,5-Tri­methyl­benzene		4.6343 (9)	3.0727 (5)	2.850	SOPLAL01	618820^*a*^
2,4,6-Tri­methyl­anilinium	SO_4_^2−^	4.486 (2)	3.3028 (14)	2.434	AZUTOF	850619^*b*^
2,4,6-Tri­methyl­anilinium	SO_4_^2−^	4.489 (3)	3.2917 (16)	2.459	AZUTOF01	733935^*c*^
2,4,6-Tri­methyl­anilinium	Br^−^	5.362 (3)	3.3138 (18)	3.886	CUCTOK	750635^*d*^
2,4,6-Tri­methyl­anilinium	I^−^	5.5497 (14)	3.4087	4.379	JEVPUW	636623^*e*^
2,4,6-Tri­methyl­anilinium	Cl^−^	4.8109 (17)	3.4992 (9)	3.302	XIFQAF	654863^*f*^
2,4,6-Tri­methyl­anilinium	NO_3_^−^	5.3297 (17)	3.0222 (7)	3.928	YUKNUO	734678^*g*^
2,4,6-Tri­methyl­anilinium	ClO_4_^−^	5.374 (2)	3.6118 (8)	3.980	YUKPAW	734679^*g*^
2,4,6-Tri­methyl­anilinium	ClO_4_^−^	5.526 (11)	3.958 (10)	3.857	YUKPAW01	865148^*h*^
2,4,6-Tri­methyl­anilinium	ClO_4_^−^	5.340 (3)	3.6060 (17)	3.939	YUKPAW02	865149^*h*^

References: (*a*) Ibberson *et al.* (2007 ▸); (*b*) Rong (2011 ▸); (*c*) Kapoor *et al.* (2010*a* ▸); (*d*) Cui & Xu (2009 ▸); (*e*) Lemmerer & Billing (2007 ▸); (*f*) Long *et al.* (2007 ▸); (*g*) Kapoor *et al.* (2010*b* ▸); (*h*) Zhang *et al.* (2012 ▸).

**Table 4:** Hydrogen-bond geometry (Å, °) for **2** [Chem scheme1]

*D*—H⋯*A*	*D*—H	H⋯*A*	*D*⋯*A*	*D*—H⋯*A*
C11—H11*A*⋯O1^i^	0.98	2.56	3.477 (2)	155
C12—H12*A*⋯F2^ii^	0.98	2.69	3.3859 (18)	129
C13—H13*A*⋯O1^i^	0.98	2.45	3.3789 (18)	159
C13—H13*B*⋯O2^iii^	0.98	2.29	3.2377 (18)	162
C13—H13*C*⋯O3	0.98	2.52	3.1723 (17)	124

Symmetry codes: (i) 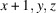 ; (ii) 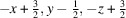 ; (iii) 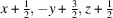 .

**Table 5:** Experimental details

	**1**	**2**
Crystal data		
Chemical formula	[C_10_H_16_N^+^][CF_3_O_3_S^−^]	[C_13_H_20_N^+^][CF_3_O_3_S^−^]
*M*_r_	299.31	339.37
Crystal system, space group	Monoclinic, *P*2_1_/*n*	Monoclinic, *P*2_1_/*n*
Temperature (K)	150	150
*a*, *b*, *c* (Å)	8.5194 (7), 18.1257 (13), 9.0875 (8)	6.8580 (4), 19.4619 (12), 12.6131 (7)
β (°)	105.106 (3)	102.024 (2)
*V* (Å^3^)	1354.80 (19)	1646.53 (17)
*Z*	4	4
Radiation type	Mo *K*α	Mo *K*α
μ (mm^−1^)	0.28	0.24
Crystal size (mm)	0.45 × 0.43 × 0.21	0.45 × 0.43 × 0.32

Data collection		
Diffractometer	Bruker AXS D8 Quest	Bruker AXS D8 Quest
Absorption correction	Multi-scan (*SADABS*; Krause *et al.*, 2015 ▸)	Multi-scan (*SADABS*; Krause *et al.*, 2015 ▸)
*T*_min_, *T*_max_	0.559, 0.747	0.662, 0.747
No. of measured, independent and observed [*I* > 2σ(*I*)] reflections	47829, 5186, 3572	35093, 6151, 4836
*R*_int_	0.093	0.040
(sin θ/λ)_max_ (Å^−1^)	0.772	0.770

Refinement		
*R*[*F* ^2^ > 2σ(*F* ^2^)], *wR*(*F* ^2^), *S*	0.047, 0.133, 1.03	0.045, 0.136, 1.04
No. of reflections	5186	6151
No. of parameters	176	205
H-atom treatment	H-atom parameters constrained	H-atom parameters constrained
Δρ_max_, Δρ_min_ (e Å^−3^)	0.42, −0.56	0.63, −0.41

Computer programs: *APEX4* and *SAINT* (Bruker, 2022 ▸), *SHELXT* (Sheldrick, 2015*a* ▸), *SHELXL2019/2* (Sheldrick, 2015*b* ▸), *ShelXle* (Hübschle *et al.*, 2011 ▸), *Mercury* (Macrae *et al.*, 2020 ▸), and *publCIF* (Westrip, 2010 ▸).

## supplementary crystallographic information

### N,2,4,6-Tetramethylanilinium trifluoromethanesulfonate (1) Crystal data

**Table:**

C_10_H_16_N^+^·CF_3_O_3_S^−^	*F*(000) = 624
*M**_r_* = 299.31	*D*_x_ = 1.467 Mg m^−^^3^
Monoclinic, *P*2_1_/*n*	Mo *K*α radiation, λ = 0.71073 Å
*a* = 8.5194 (7) Å	Cell parameters from 9989 reflections
*b* = 18.1257 (13) Å	θ = 2.6–32.7°
*c* = 9.0875 (8) Å	µ = 0.28 mm^−^^1^
β = 105.106 (3)°	*T* = 150 K
*V* = 1354.80 (19) Å^3^	Plate, colourless
*Z* = 4	0.45 × 0.43 × 0.21 mm

### N,2,4,6-Tetramethylanilinium trifluoromethanesulfonate (1) Data collection

**Table:**

Bruker AXS D8 Quest diffractometer	5186 independent reflections
Radiation source: fine focus sealed tube X-ray source	3572 reflections with *I* > 2σ(*I*)
Triumph curved graphite crystal monochromator	*R*_int_ = 0.093
Detector resolution: 7.4074 pixels mm^-1^	θ_max_ = 33.3°, θ_min_ = 2.6°
ω and phi scans	*h* = −13→12
Absorption correction: multi-scan (SADABS; Krause *et al*., 2015)	*k* = −27→27
*T*_min_ = 0.559, *T*_max_ = 0.747	*l* = −13→14
47829 measured reflections	

### N,2,4,6-Tetramethylanilinium trifluoromethanesulfonate (1) Refinement

**Table:**

Refinement on *F*^2^	Primary atom site location: dual
Least-squares matrix: full	Secondary atom site location: difference Fourier map
*R*[*F*^2^ > 2σ(*F*^2^)] = 0.047	Hydrogen site location: inferred from neighbouring sites
*wR*(*F*^2^) = 0.133	H-atom parameters constrained
*S* = 1.03	*w* = 1/[σ^2^(*F*_o_^2^) + (0.0667*P*)^2^ + 0.3108*P*] where *P* = (*F*_o_^2^ + 2*F*_c_^2^)/3
5186 reflections	(Δ/σ)_max_ = 0.001
176 parameters	Δρ_max_ = 0.42 e Å^−^^3^
0 restraints	Δρ_min_ = −0.56 e Å^−^^3^

### N,2,4,6-Tetramethylanilinium trifluoromethanesulfonate (1) Special details

**Table:**

Geometry. All esds (except the esd in the dihedral angle between two l.s. planes) are estimated using the full covariance matrix. The cell esds are taken into account individually in the estimation of esds in distances, angles and torsion angles; correlations between esds in cell parameters are only used when they are defined by crystal symmetry. An approximate (isotropic) treatment of cell esds is used for estimating esds involving l.s. planes.

### N,2,4,6-Tetramethylanilinium trifluoromethanesulfonate (1) Fractional atomic coordinates and isotropic or equivalent isotropic displacement parameters (Å^2^)

**Table:**

	*x*	*y*	*z*	*U*_iso_*/*U*_eq_	
S1	0.26254 (4)	0.70859 (2)	0.03063 (4)	0.02587 (10)	
F1	0.06186 (16)	0.66503 (9)	−0.22410 (12)	0.0730 (4)	
F2	−0.00260 (12)	0.62874 (6)	−0.02375 (13)	0.0491 (3)	
F3	0.19455 (15)	0.57716 (7)	−0.08796 (18)	0.0703 (4)	
O1	0.31540 (15)	0.67224 (7)	0.17559 (12)	0.0431 (3)	
O2	0.16299 (15)	0.77239 (7)	0.03049 (17)	0.0469 (3)	
O3	0.38537 (14)	0.71728 (7)	−0.04960 (13)	0.0380 (3)	
N1	0.38672 (14)	0.64116 (6)	0.48425 (12)	0.0243 (2)	
H1A	0.358888	0.636006	0.381079	0.029*	
H1B	0.493405	0.654345	0.513906	0.029*	
C1	0.36660 (15)	0.56949 (7)	0.55382 (14)	0.0221 (2)	
C2	0.24275 (15)	0.52245 (7)	0.47553 (14)	0.0229 (2)	
C3	0.21912 (17)	0.45701 (7)	0.54737 (15)	0.0252 (2)	
H3	0.136131	0.424047	0.496070	0.030*	
C4	0.31396 (16)	0.43847 (7)	0.69272 (15)	0.0252 (3)	
C5	0.43919 (16)	0.48619 (8)	0.76333 (14)	0.0257 (3)	
H5	0.507495	0.473066	0.860156	0.031*	
C6	0.46785 (15)	0.55238 (7)	0.69717 (14)	0.0236 (2)	
C7	0.13840 (17)	0.53992 (8)	0.31822 (15)	0.0282 (3)	
H7A	0.204815	0.537779	0.245017	0.042*	
H7B	0.092515	0.589532	0.317709	0.042*	
H7C	0.050034	0.503828	0.289669	0.042*	
C8	0.27871 (19)	0.37021 (8)	0.77291 (17)	0.0318 (3)	
H8A	0.219874	0.334557	0.697413	0.048*	
H8B	0.212107	0.383332	0.842138	0.048*	
H8C	0.381199	0.348317	0.831371	0.048*	
C9	0.60280 (17)	0.60290 (8)	0.77978 (16)	0.0305 (3)	
H9A	0.673863	0.614143	0.713537	0.046*	
H9B	0.666105	0.578630	0.872717	0.046*	
H9C	0.556113	0.648773	0.806764	0.046*	
C10	0.2861 (2)	0.70206 (8)	0.52615 (18)	0.0338 (3)	
H10A	0.304250	0.747882	0.475748	0.051*	
H10B	0.317801	0.709186	0.636870	0.051*	
H10C	0.170738	0.688743	0.493086	0.051*	
C11	0.1221 (2)	0.64161 (10)	−0.08269 (18)	0.0374 (3)	

### N,2,4,6-Tetramethylanilinium trifluoromethanesulfonate (1) Atomic displacement parameters (Å^2^)

**Table:**

	*U*^11^	*U*^22^	*U*^33^	*U*^12^	*U*^13^	*U*^23^
S1	0.02667 (16)	0.03106 (18)	0.02113 (15)	0.00514 (13)	0.00846 (11)	0.00329 (12)
F1	0.0665 (8)	0.1198 (12)	0.0243 (5)	−0.0232 (8)	−0.0031 (5)	0.0009 (6)
F2	0.0354 (5)	0.0635 (7)	0.0509 (6)	−0.0111 (5)	0.0155 (4)	−0.0054 (5)
F3	0.0570 (7)	0.0532 (7)	0.1026 (11)	−0.0022 (6)	0.0240 (7)	−0.0397 (7)
O1	0.0452 (6)	0.0557 (7)	0.0246 (5)	−0.0077 (6)	0.0022 (4)	0.0128 (5)
O2	0.0396 (6)	0.0398 (6)	0.0647 (9)	0.0121 (5)	0.0197 (6)	−0.0029 (6)
O3	0.0346 (5)	0.0491 (7)	0.0350 (6)	0.0031 (5)	0.0174 (5)	0.0045 (5)
N1	0.0262 (5)	0.0269 (5)	0.0212 (5)	−0.0026 (4)	0.0084 (4)	−0.0013 (4)
C1	0.0247 (5)	0.0245 (6)	0.0192 (5)	0.0002 (5)	0.0095 (4)	−0.0013 (4)
C2	0.0228 (5)	0.0280 (6)	0.0187 (5)	0.0001 (5)	0.0072 (4)	−0.0007 (4)
C3	0.0277 (6)	0.0268 (6)	0.0226 (5)	−0.0015 (5)	0.0092 (5)	−0.0013 (5)
C4	0.0294 (6)	0.0258 (6)	0.0231 (6)	0.0043 (5)	0.0118 (5)	0.0014 (5)
C5	0.0275 (6)	0.0315 (7)	0.0190 (5)	0.0041 (5)	0.0079 (4)	−0.0001 (5)
C6	0.0236 (5)	0.0300 (6)	0.0185 (5)	0.0014 (5)	0.0076 (4)	−0.0037 (4)
C7	0.0278 (6)	0.0336 (7)	0.0217 (6)	−0.0034 (5)	0.0037 (5)	0.0027 (5)
C8	0.0378 (7)	0.0307 (7)	0.0290 (7)	0.0030 (6)	0.0124 (6)	0.0069 (5)
C9	0.0302 (7)	0.0364 (7)	0.0237 (6)	−0.0027 (6)	0.0049 (5)	−0.0046 (5)
C10	0.0411 (8)	0.0264 (7)	0.0382 (8)	0.0043 (6)	0.0176 (6)	0.0005 (6)
C11	0.0352 (7)	0.0486 (9)	0.0288 (7)	0.0009 (7)	0.0088 (6)	−0.0056 (6)

### N,2,4,6-Tetramethylanilinium trifluoromethanesulfonate (1) Geometric parameters (Å, º)

**Table:**

S1—O3	1.4315 (11)	C4—C5	1.393 (2)
S1—O2	1.4339 (12)	C4—C8	1.5052 (19)
S1—O1	1.4364 (11)	C5—C6	1.3918 (19)
S1—C11	1.8230 (17)	C5—H5	0.9500
F1—C11	1.3234 (19)	C6—C9	1.5080 (19)
F2—C11	1.3293 (19)	C7—H7A	0.9800
F3—C11	1.328 (2)	C7—H7B	0.9800
N1—C1	1.4742 (17)	C7—H7C	0.9800
N1—C10	1.5062 (18)	C8—H8A	0.9800
N1—H1A	0.9100	C8—H8B	0.9800
N1—H1B	0.9100	C8—H8C	0.9800
C1—C6	1.3970 (18)	C9—H9A	0.9800
C1—C2	1.3983 (18)	C9—H9B	0.9800
C2—C3	1.3935 (18)	C9—H9C	0.9800
C2—C7	1.5073 (18)	C10—H10A	0.9800
C3—C4	1.3980 (18)	C10—H10B	0.9800
C3—H3	0.9500	C10—H10C	0.9800
			
O3—S1—O2	114.93 (8)	C2—C7—H7A	109.5
O3—S1—O1	114.93 (7)	C2—C7—H7B	109.5
O2—S1—O1	114.51 (8)	H7A—C7—H7B	109.5
O3—S1—C11	104.16 (7)	C2—C7—H7C	109.5
O2—S1—C11	103.63 (8)	H7A—C7—H7C	109.5
O1—S1—C11	102.43 (7)	H7B—C7—H7C	109.5
C1—N1—C10	113.57 (10)	C4—C8—H8A	109.5
C1—N1—H1A	108.9	C4—C8—H8B	109.5
C10—N1—H1A	108.9	H8A—C8—H8B	109.5
C1—N1—H1B	108.9	C4—C8—H8C	109.5
C10—N1—H1B	108.9	H8A—C8—H8C	109.5
H1A—N1—H1B	107.7	H8B—C8—H8C	109.5
C6—C1—C2	122.66 (12)	C6—C9—H9A	109.5
C6—C1—N1	118.88 (11)	C6—C9—H9B	109.5
C2—C1—N1	118.41 (11)	H9A—C9—H9B	109.5
C3—C2—C1	117.56 (12)	C6—C9—H9C	109.5
C3—C2—C7	120.11 (12)	H9A—C9—H9C	109.5
C1—C2—C7	122.32 (12)	H9B—C9—H9C	109.5
C2—C3—C4	121.93 (12)	N1—C10—H10A	109.5
C2—C3—H3	119.0	N1—C10—H10B	109.5
C4—C3—H3	119.0	H10A—C10—H10B	109.5
C5—C4—C3	118.07 (12)	N1—C10—H10C	109.5
C5—C4—C8	120.84 (12)	H10A—C10—H10C	109.5
C3—C4—C8	121.06 (13)	H10B—C10—H10C	109.5
C6—C5—C4	122.39 (12)	F1—C11—F3	108.27 (15)
C6—C5—H5	118.8	F1—C11—F2	107.40 (14)
C4—C5—H5	118.8	F3—C11—F2	106.73 (15)
C5—C6—C1	117.33 (12)	F1—C11—S1	111.49 (13)
C5—C6—C9	120.35 (12)	F3—C11—S1	111.33 (12)
C1—C6—C9	122.33 (12)	F2—C11—S1	111.41 (11)
			
C10—N1—C1—C6	−89.12 (15)	C2—C1—C6—C5	−1.40 (18)
C10—N1—C1—C2	88.69 (14)	N1—C1—C6—C5	176.31 (11)
C6—C1—C2—C3	1.63 (19)	C2—C1—C6—C9	179.04 (12)
N1—C1—C2—C3	−176.09 (11)	N1—C1—C6—C9	−3.25 (18)
C6—C1—C2—C7	−177.50 (12)	O3—S1—C11—F1	−58.65 (14)
N1—C1—C2—C7	4.78 (18)	O2—S1—C11—F1	61.92 (14)
C1—C2—C3—C4	0.40 (19)	O1—S1—C11—F1	−178.71 (12)
C7—C2—C3—C4	179.54 (12)	O3—S1—C11—F3	62.38 (14)
C2—C3—C4—C5	−2.52 (19)	O2—S1—C11—F3	−177.05 (13)
C2—C3—C4—C8	175.57 (12)	O1—S1—C11—F3	−57.68 (14)
C3—C4—C5—C6	2.77 (19)	O3—S1—C11—F2	−178.61 (11)
C8—C4—C5—C6	−175.32 (12)	O2—S1—C11—F2	−58.04 (14)
C4—C5—C6—C1	−0.87 (19)	O1—S1—C11—F2	61.33 (13)
C4—C5—C6—C9	178.69 (12)		

### N,2,4,6-Tetramethylanilinium trifluoromethanesulfonate (1) Hydrogen-bond geometry (Å, º)

**Table:**

*D*—H···*A*	*D*—H	H···*A*	*D*···*A*	*D*—H···*A*
N1—H1*A*···O1	0.91	1.92	2.7687 (16)	154
N1—H1*B*···O2^i^	0.91	1.94	2.7669 (16)	150
C8—H8*C*···O1^ii^	0.98	2.63	3.453 (2)	142
C8—H8*C*···O3^ii^	0.98	2.69	3.6438 (19)	164
C8—H8*A*···O3^iii^	0.98	2.55	3.504 (2)	165
C9—H9*A*···O2^i^	0.98	2.63	3.333 (2)	129

Symmetry codes: (i) *x*+1/2, −*y*+3/2, *z*+1/2; (ii) −*x*+1, −*y*+1, −*z*+1; (iii) −*x*+1/2, *y*−1/2, −*z*+1/2.

### N-Isopropylidene-N,2,4,6-tetramethylanilinium trifluoromethanesulfonate (2) Crystal data

**Table:**

C_13_H_20_N^+^·CF_3_O_3_S^−^	*F*(000) = 712
*M**_r_* = 339.37	*D*_x_ = 1.369 Mg m^−^^3^
Monoclinic, *P*2_1_/*n*	Mo *K*α radiation, λ = 0.71073 Å
*a* = 6.8580 (4) Å	Cell parameters from 9901 reflections
*b* = 19.4619 (12) Å	θ = 2.7–33.1°
*c* = 12.6131 (7) Å	µ = 0.24 mm^−^^1^
β = 102.024 (2)°	*T* = 150 K
*V* = 1646.53 (17) Å^3^	Block, colourless
*Z* = 4	0.45 × 0.43 × 0.32 mm

### N-Isopropylidene-N,2,4,6-tetramethylanilinium trifluoromethanesulfonate (2) Data collection

**Table:**

Bruker AXS D8 Quest diffractometer	6151 independent reflections
Radiation source: fine focus sealed tube X-ray source	4836 reflections with *I* > 2σ(*I*)
Triumph curved graphite crystal monochromator	*R*_int_ = 0.040
Detector resolution: 7.4074 pixels mm^-1^	θ_max_ = 33.2°, θ_min_ = 2.0°
ω and phi scans	*h* = −10→10
Absorption correction: multi-scan (SADABS; Krause *et al*., 2015)	*k* = −29→28
*T*_min_ = 0.662, *T*_max_ = 0.747	*l* = −19→19
35093 measured reflections	

### N-Isopropylidene-N,2,4,6-tetramethylanilinium trifluoromethanesulfonate (2) Refinement

**Table:**

Refinement on *F*^2^	0 restraints
Least-squares matrix: full	Hydrogen site location: inferred from neighbouring sites
*R*[*F*^2^ > 2σ(*F*^2^)] = 0.045	H-atom parameters constrained
*wR*(*F*^2^) = 0.136	*w* = 1/[σ^2^(*F*_o_^2^) + (0.0647*P*)^2^ + 0.6525*P*] where *P* = (*F*_o_^2^ + 2*F*_c_^2^)/3
*S* = 1.04	(Δ/σ)_max_ = 0.036
6151 reflections	Δρ_max_ = 0.63 e Å^−^^3^
205 parameters	Δρ_min_ = −0.41 e Å^−^^3^

### N-Isopropylidene-N,2,4,6-tetramethylanilinium trifluoromethanesulfonate (2) Special details

**Table:**

Geometry. All esds (except the esd in the dihedral angle between two l.s. planes) are estimated using the full covariance matrix. The cell esds are taken into account individually in the estimation of esds in distances, angles and torsion angles; correlations between esds in cell parameters are only used when they are defined by crystal symmetry. An approximate (isotropic) treatment of cell esds is used for estimating esds involving l.s. planes.

### N-Isopropylidene-N,2,4,6-tetramethylanilinium trifluoromethanesulfonate (2) Fractional atomic coordinates and isotropic or equivalent isotropic displacement parameters (Å^2^)

**Table:**

	*x*	*y*	*z*	*U*_iso_*/*U*_eq_	
S1	0.15479 (4)	0.79780 (2)	0.61391 (2)	0.02550 (8)	
F1	0.3761 (3)	0.86287 (8)	0.77561 (9)	0.0851 (5)	
F2	0.2530 (2)	0.92744 (5)	0.64188 (11)	0.0664 (3)	
F3	0.49502 (18)	0.86146 (8)	0.63158 (12)	0.0734 (4)	
O1	−0.02107 (18)	0.81355 (7)	0.65353 (12)	0.0494 (3)	
O2	0.1341 (2)	0.80438 (7)	0.49895 (9)	0.0507 (3)	
O3	0.25615 (18)	0.73657 (6)	0.66054 (11)	0.0496 (3)	
N1	0.86535 (15)	0.64144 (5)	0.77099 (8)	0.02325 (18)	
C1	0.81204 (17)	0.57568 (6)	0.71732 (9)	0.0235 (2)	
C2	0.73919 (18)	0.52358 (6)	0.77498 (10)	0.0266 (2)	
C3	0.68216 (19)	0.46217 (6)	0.72058 (12)	0.0310 (3)	
H3	0.631492	0.426053	0.757740	0.037*	
C4	0.69727 (19)	0.45226 (7)	0.61361 (12)	0.0318 (3)	
C5	0.7696 (2)	0.50569 (7)	0.55946 (11)	0.0320 (3)	
H5	0.779749	0.499239	0.486153	0.038*	
C6	0.82752 (19)	0.56836 (6)	0.60949 (10)	0.0269 (2)	
C7	0.7275 (2)	0.53217 (8)	0.89215 (12)	0.0370 (3)	
H7A	0.675490	0.577958	0.903002	0.055*	
H7B	0.860854	0.526986	0.937972	0.055*	
H7C	0.638530	0.497155	0.911733	0.055*	
C8	0.6377 (2)	0.38445 (8)	0.55818 (16)	0.0450 (4)	
H8A	0.613907	0.390669	0.479424	0.067*	
H8B	0.515577	0.367745	0.578344	0.067*	
H8C	0.744876	0.350912	0.580769	0.067*	
C9	0.9047 (2)	0.62569 (7)	0.54963 (12)	0.0365 (3)	
H9A	0.898812	0.611766	0.474357	0.055*	
H9B	1.043117	0.635892	0.584635	0.055*	
H9C	0.822601	0.666766	0.550892	0.055*	
C10	1.03905 (18)	0.65257 (6)	0.83181 (10)	0.0274 (2)	
C11	1.0819 (2)	0.71841 (7)	0.89199 (12)	0.0368 (3)	
H11A	1.071297	0.756521	0.840322	0.055*	
H11B	1.217001	0.717058	0.936743	0.055*	
H11C	0.985468	0.725038	0.938559	0.055*	
C12	1.2004 (2)	0.60056 (8)	0.84344 (15)	0.0411 (3)	
H12A	1.148345	0.558503	0.805015	0.062*	
H12B	1.249569	0.590234	0.920420	0.062*	
H12C	1.309884	0.618616	0.812571	0.062*	
C13	0.70636 (19)	0.69461 (6)	0.75334 (11)	0.0293 (2)	
H13A	0.758175	0.737207	0.728243	0.044*	
H13B	0.663458	0.703278	0.821535	0.044*	
H13C	0.592598	0.678426	0.698583	0.044*	
C14	0.3300 (3)	0.86552 (8)	0.66949 (12)	0.0387 (3)	

### N-Isopropylidene-N,2,4,6-tetramethylanilinium trifluoromethanesulfonate (2) Atomic displacement parameters (Å^2^)

**Table:**

	*U*^11^	*U*^22^	*U*^33^	*U*^12^	*U*^13^	*U*^23^
S1	0.02328 (14)	0.02674 (14)	0.02618 (14)	−0.00154 (10)	0.00446 (10)	−0.00088 (10)
F1	0.1245 (13)	0.0883 (10)	0.0321 (5)	−0.0545 (9)	−0.0077 (6)	−0.0097 (6)
F2	0.0969 (10)	0.0280 (5)	0.0795 (8)	−0.0120 (5)	0.0304 (7)	−0.0050 (5)
F3	0.0431 (6)	0.0875 (9)	0.0941 (10)	−0.0345 (6)	0.0248 (6)	−0.0337 (8)
O1	0.0396 (6)	0.0469 (6)	0.0695 (8)	−0.0009 (5)	0.0291 (6)	−0.0024 (6)
O2	0.0612 (8)	0.0616 (8)	0.0282 (5)	−0.0198 (6)	0.0068 (5)	−0.0046 (5)
O3	0.0405 (6)	0.0309 (5)	0.0708 (8)	0.0043 (4)	−0.0036 (6)	0.0016 (5)
N1	0.0232 (4)	0.0196 (4)	0.0266 (4)	0.0017 (3)	0.0043 (3)	0.0010 (3)
C1	0.0221 (5)	0.0191 (4)	0.0286 (5)	−0.0006 (4)	0.0036 (4)	0.0011 (4)
C2	0.0235 (5)	0.0235 (5)	0.0324 (5)	0.0009 (4)	0.0050 (4)	0.0063 (4)
C3	0.0259 (6)	0.0206 (5)	0.0461 (7)	−0.0013 (4)	0.0063 (5)	0.0071 (5)
C4	0.0265 (6)	0.0204 (5)	0.0466 (7)	−0.0025 (4)	0.0032 (5)	−0.0026 (5)
C5	0.0351 (6)	0.0269 (6)	0.0335 (6)	−0.0043 (5)	0.0062 (5)	−0.0046 (5)
C6	0.0293 (6)	0.0228 (5)	0.0288 (5)	−0.0032 (4)	0.0069 (4)	0.0002 (4)
C7	0.0401 (7)	0.0387 (7)	0.0335 (6)	0.0011 (6)	0.0110 (5)	0.0099 (5)
C8	0.0404 (8)	0.0247 (6)	0.0681 (11)	−0.0077 (5)	0.0073 (7)	−0.0115 (6)
C9	0.0496 (8)	0.0298 (6)	0.0328 (6)	−0.0107 (6)	0.0145 (6)	0.0006 (5)
C10	0.0257 (5)	0.0237 (5)	0.0310 (5)	−0.0004 (4)	0.0022 (4)	0.0016 (4)
C11	0.0405 (7)	0.0300 (6)	0.0367 (6)	−0.0056 (5)	0.0004 (5)	−0.0065 (5)
C12	0.0256 (6)	0.0317 (7)	0.0604 (9)	0.0039 (5)	−0.0042 (6)	0.0033 (6)
C13	0.0266 (6)	0.0253 (5)	0.0360 (6)	0.0076 (4)	0.0066 (5)	0.0024 (4)
C14	0.0479 (8)	0.0345 (7)	0.0344 (6)	−0.0132 (6)	0.0101 (6)	−0.0067 (5)

### N-Isopropylidene-N,2,4,6-tetramethylanilinium trifluoromethanesulfonate (2) Geometric parameters (Å, º)

**Table:**

S1—O1	1.4315 (12)	C7—H7A	0.9800
S1—O2	1.4330 (12)	C7—H7B	0.9800
S1—O3	1.4419 (12)	C7—H7C	0.9800
S1—C14	1.8226 (15)	C8—H8A	0.9800
F1—C14	1.3107 (18)	C8—H8B	0.9800
F2—C14	1.333 (2)	C8—H8C	0.9800
F3—C14	1.319 (2)	C9—H9A	0.9800
N1—C10	1.2937 (16)	C9—H9B	0.9800
N1—C1	1.4584 (15)	C9—H9C	0.9800
N1—C13	1.4860 (15)	C10—C12	1.4843 (18)
C1—C6	1.3934 (17)	C10—C11	1.4871 (18)
C1—C2	1.3994 (16)	C11—H11A	0.9800
C2—C3	1.3929 (18)	C11—H11B	0.9800
C2—C7	1.5060 (19)	C11—H11C	0.9800
C3—C4	1.388 (2)	C12—H12A	0.9800
C3—H3	0.9500	C12—H12B	0.9800
C4—C5	1.3910 (19)	C12—H12C	0.9800
C4—C8	1.5095 (19)	C13—H13A	0.9800
C5—C6	1.3925 (17)	C13—H13B	0.9800
C5—H5	0.9500	C13—H13C	0.9800
C6—C9	1.5039 (18)		
			
O1—S1—O2	114.90 (9)	C4—C8—H8C	109.5
O1—S1—O3	113.84 (8)	H8A—C8—H8C	109.5
O2—S1—O3	115.11 (8)	H8B—C8—H8C	109.5
O1—S1—C14	104.29 (8)	C6—C9—H9A	109.5
O2—S1—C14	104.07 (7)	C6—C9—H9B	109.5
O3—S1—C14	102.57 (8)	H9A—C9—H9B	109.5
C10—N1—C1	122.21 (10)	C6—C9—H9C	109.5
C10—N1—C13	121.91 (11)	H9A—C9—H9C	109.5
C1—N1—C13	115.87 (10)	H9B—C9—H9C	109.5
C6—C1—C2	122.77 (11)	N1—C10—C12	121.33 (12)
C6—C1—N1	118.69 (10)	N1—C10—C11	120.42 (12)
C2—C1—N1	118.47 (11)	C12—C10—C11	118.25 (12)
C3—C2—C1	117.36 (12)	C10—C11—H11A	109.5
C3—C2—C7	120.79 (12)	C10—C11—H11B	109.5
C1—C2—C7	121.83 (12)	H11A—C11—H11B	109.5
C4—C3—C2	121.87 (12)	C10—C11—H11C	109.5
C4—C3—H3	119.1	H11A—C11—H11C	109.5
C2—C3—H3	119.1	H11B—C11—H11C	109.5
C3—C4—C5	118.67 (12)	C10—C12—H12A	109.5
C3—C4—C8	120.26 (13)	C10—C12—H12B	109.5
C5—C4—C8	121.06 (14)	H12A—C12—H12B	109.5
C4—C5—C6	121.98 (12)	C10—C12—H12C	109.5
C4—C5—H5	119.0	H12A—C12—H12C	109.5
C6—C5—H5	119.0	H12B—C12—H12C	109.5
C5—C6—C1	117.34 (11)	N1—C13—H13A	109.5
C5—C6—C9	121.29 (12)	N1—C13—H13B	109.5
C1—C6—C9	121.37 (11)	H13A—C13—H13B	109.5
C2—C7—H7A	109.5	N1—C13—H13C	109.5
C2—C7—H7B	109.5	H13A—C13—H13C	109.5
H7A—C7—H7B	109.5	H13B—C13—H13C	109.5
C2—C7—H7C	109.5	F1—C14—F3	108.99 (16)
H7A—C7—H7C	109.5	F1—C14—F2	107.49 (14)
H7B—C7—H7C	109.5	F3—C14—F2	106.43 (14)
C4—C8—H8A	109.5	F1—C14—S1	111.32 (11)
C4—C8—H8B	109.5	F3—C14—S1	111.33 (11)
H8A—C8—H8B	109.5	F2—C14—S1	111.08 (12)
			
C10—N1—C1—C6	−97.12 (14)	N1—C1—C6—C5	−178.02 (11)
C13—N1—C1—C6	84.04 (14)	C2—C1—C6—C9	179.42 (13)
C10—N1—C1—C2	85.64 (15)	N1—C1—C6—C9	2.30 (18)
C13—N1—C1—C2	−93.20 (13)	C1—N1—C10—C12	4.98 (19)
C6—C1—C2—C3	0.47 (18)	C13—N1—C10—C12	−176.25 (13)
N1—C1—C2—C3	177.60 (10)	C1—N1—C10—C11	−175.12 (12)
C6—C1—C2—C7	178.81 (12)	C13—N1—C10—C11	3.65 (19)
N1—C1—C2—C7	−4.06 (18)	O1—S1—C14—F1	63.94 (16)
C1—C2—C3—C4	0.35 (19)	O2—S1—C14—F1	−175.28 (14)
C7—C2—C3—C4	−178.00 (12)	O3—S1—C14—F1	−55.02 (16)
C2—C3—C4—C5	−0.7 (2)	O1—S1—C14—F3	−174.22 (13)
C2—C3—C4—C8	178.79 (13)	O2—S1—C14—F3	−53.45 (15)
C3—C4—C5—C6	0.2 (2)	O3—S1—C14—F3	66.82 (14)
C8—C4—C5—C6	−179.25 (13)	O1—S1—C14—F2	−55.81 (13)
C4—C5—C6—C1	0.5 (2)	O2—S1—C14—F2	64.97 (13)
C4—C5—C6—C9	−179.79 (14)	O3—S1—C14—F2	−174.77 (11)
C2—C1—C6—C5	−0.90 (19)		

### N-Isopropylidene-N,2,4,6-tetramethylanilinium trifluoromethanesulfonate (2) Hydrogen-bond geometry (Å, º)

**Table:**

*D*—H···*A*	*D*—H	H···*A*	*D*···*A*	*D*—H···*A*
C11—H11*A*···O1^i^	0.98	2.56	3.477 (2)	155
C12—H12*A*···F2^ii^	0.98	2.69	3.3859 (18)	129
C13—H13*A*···O1^i^	0.98	2.45	3.3789 (18)	159
C13—H13*B*···O2^iii^	0.98	2.29	3.2377 (18)	162
C13—H13*C*···O3	0.98	2.52	3.1723 (17)	124

Symmetry codes: (i) *x*+1, *y*, *z*; (ii) −*x*+3/2, *y*−1/2, −*z*+3/2; (iii) *x*+1/2, −*y*+3/2, *z*+1/2.
